# Clinical *CYP2D6* Genotyping to Personalize Adjuvant Tamoxifen Treatment in ER-Positive Breast Cancer Patients: Current Status of a Controversy

**DOI:** 10.3390/cancers13040771

**Published:** 2021-02-12

**Authors:** Tessa A. M. Mulder, Mirjam de With, Marzia del Re, Romano Danesi, Ron H. J. Mathijssen, Ron H. N. van Schaik

**Affiliations:** 1 Department of Clinical Chemistry, Erasmus MC University Hospital, Wytemaweg 80, 3015CN Rotterdam, The Netherlands; t.mulder@erasmusmc.nl (T.A.M.M.); m.dewith@erasmusmc.nl (M.d.W.); marzia.delre@gmail.com (M.d.R.); romano.danesi@med.unipi.it (R.D.); 2 Department of Medical Oncology, Erasmus MC Cancer Institute, Erasmus MC, Wytemaweg 80, 3015CN Rotterdam, The Netherlands; a.mathijssen@erasmusmc.nl; 3 Clinical Pharmacology and Pharmacogenetics Unit, Department of Clinical and Experimental Medicine, University of Pisa, 55, Via Roma, 56126 Pisa, Italy

**Keywords:** tamoxifen, Pharmacogenetics, PGx, CYP2D6, Cytochrome P450 2D6, genotyping, tamoxifen treatment, review

## Abstract

**Simple Summary:**

Tamoxifen is an important adjuvant endocrine therapy in estrogen receptor (ER)-positive breast cancer patients. It is mainly catalyzed by the enzyme CYP2D6 into the most active metabolite endoxifen. Genetic variation in the *CYP2D6* gene influences endoxifen formation and thereby potentially therapy outcome. However, the association between *CYP2D6* genotype and clinical outcome on tamoxifen is still under debate, as contradictory outcomes have been published. This review describes the latest insights in both *CYP2D6* genotype and endoxifen concentrations, as well *CYP2D6* genotype and clinical outcome, from 2018 to 2020.

**Abstract:**

Tamoxifen is a major option for adjuvant endocrine treatment in estrogen receptor (ER) positive breast cancer patients. The conversion of the prodrug tamoxifen into the most active metabolite endoxifen is mainly catalyzed by the enzyme cytochrome P450 2D6 (CYP2D6). Genetic variation in the *CYP2D6* gene leads to altered enzyme activity, which influences endoxifen formation and thereby potentially therapy outcome. The association between genetically compromised CYP2D6 activity and low endoxifen plasma concentrations is generally accepted, and it was shown that tamoxifen dose increments in compromised patients resulted in higher endoxifen concentrations. However, the correlation between *CYP2D6* genotype and clinical outcome is still under debate. This has led to genotype-based tamoxifen dosing recommendations by the Clinical Pharmacogenetic Implementation Consortium (CPIC) in 2018, whereas in 2019, the European Society of Medical Oncology (ESMO) discouraged the use of *CYP2D6* genotyping in clinical practice for tamoxifen therapy. This paper describes the latest developments on *CYP2D6* genotyping in relation to endoxifen plasma concentrations and tamoxifen-related clinical outcome. Therefore, we focused on Pharmacogenetic publications from 2018 (CPIC publication) to 2021 in order to shed a light on the current status of this debate.

## 1. Introduction

For many years, tamoxifen has been known as the most important adjuvant endocrine treatment in patients with estrogen receptor (ER) positive breast cancer [1,2]. It is a selective estrogen receptor modulator (SERM), which inhibits tumor growth and promotes apoptosis in ER-positive tumors [3], resulting in a reduced risk of recurrence and death from breast cancer [4]. Tamoxifen is metabolized into its most active antiestrogenic metabolite endoxifen, predominantly by cytochrome P450 2D6 (CYP2D6). Despite high efficacy, a wide variability in the response of individuals to standard doses of tamoxifen is seen [4]. Factors influencing drug responses such as gender, age, obesity, drug–drug interactions, drug–food interactions, comorbidity, liver and renal function, pregnancy, circadian rhythm, as well as genetic factors could possibly explain this wide variability [5,6,7,8,9,10].

As genetic variation in *CYP2D6* leads to altered enzyme activity and thereby potentially to an affected efficacy [5], pharmacogenetic (PGx) testing could play a major role in optimizing tamoxifen treatment. Nowadays, PGx testing is available for an increasing number of drugs, mainly in psychiatric but also for cardiologic and oncologic applications. However, only a small number of drugs are considered to require upfront genotyping, such as HLA-B*5701 genotyping for abacavir, *CYP2C19* genotyping for clopidogrel, and *DPYD* testing for fluoropyrimidines treatment [11,12,13]. The challenge of PGx testing is to obtain actionable information of genetic variants and their influence on outcome. Experts differ in their interpretation of published evidence and their recommendations [5]. This also applies to tamoxifen. Whereas the first studies on *CYP2D6* genotyping for optimizing tamoxifen therapy based on pharmacokinetic studies [14] or outcome [15,16] were published in 2005, its clinical implementation is still being debated [17,18]. The controversy was most prominently seen in 2012. At that time, two studies were published, and both concluded there was no significant association between *CYP2D6* genotype and outcome in breast cancer patients treated with adjuvant tamoxifen therapy [19,20]. This triggered several researchers with opposite visions, leading to a correspondence as published in the JNCI [21,22,23,24]. In 2013, several meta-analyses were performed in order to answer the question of whether *CYP2D6* status affects clinical outcomes in tamoxifen therapy [25,26,27,28,29]. However, also these findings yielded contradictory results. In 2018, the Clinical Pharmacogenetics Implementation Consortium (CPIC) published a recommendation on *CYP2D6* genotyping for guiding adjuvant tamoxifen therapy, thus supporting the importance of the *CYP2D6* genotype in tamoxifen therapy. However, the European Society for Medical Oncology (ESMO) indicated in a publication in 2019 that according to their view, there was no place for *CYP2D6* genotyping in a clinical setting [18].

According to predictions, over 3 million women will be diagnosed with breast cancer in 2040 [30], which triggers the strong need for optimizing tamoxifen treatment. Therefore, the primary objective of this review is to discuss new data on *CYP2D6* genotyping in breast cancer patients treated with tamoxifen. In addition, we included some other factors influencing plasma concentrations of tamoxifen and its metabolites. We searched for relevant studies published from 2018 (publication CPIC) to 2021 on *CYP2D6* genotype in relation to endoxifen levels and tamoxifen-related clinical outcome.

## 2. Tamoxifen Metabolism

**Figure 1:**
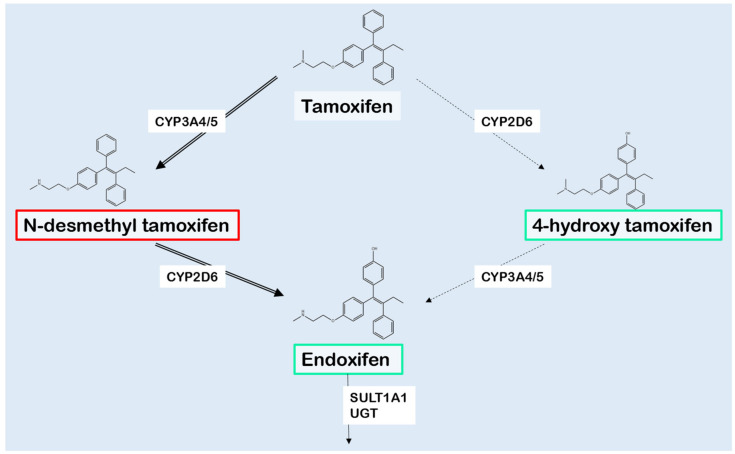
Simplistic representation of the main biotransformation of tamoxifen and its metabolites. The generation of N-desmethyltamoxifen (NDM-TAM) is predominantly catalyzed by CYP3A4/5, whereas especially CYP2D6 is responsible for the formation of 4-hydroxytamoxifen (4OH-TAM) and endoxifen. The activity of metabolites is shown using red to indicate for inactivity and green for activity. The various metabolites are inactivated by UGTs and SULTs, mainly isoform SULT1A1. Abbreviations: CYP: Cytochrome P450 isoenzymes, UGT: UDP-glucuronosyltransferase, SULT: sulfotransferase isoenzyme. Figure based on Jin, et al. [14].

Tamoxifen is a prodrug, which is converted into multiple derivatives by phase I enzymes. Among these, 4-hydroxytamoxifen (4OH-TAM) and endoxifen show the strongest affinity with the ER [2,31]. As the plasma concentration of endoxifen is 6 to 12 times higher compared with 4OH-TAM, and endoxifen has the lowest IC50 at the ER, endoxifen is considered the major active tamoxifen metabolite [2,31]. Tamoxifen is *N*-demethylated, predominantly by CYP3A4 and CYP3A5, resulting in inactive *N*-desmethyltamoxifen (NDM-TAM) (Figure 1). 4-hydroxylation of NDM-TAM is nearly exclusively performed by CYP2D6, resulting in the formation of 4-hydroxy-*N*-desmethyltamoxifen, also known as endoxifen [2,31]. As shown in Figure 1, demethylation and hydroxylation can also occur in the opposite order, resulting a different intermediate metabolite called 4-hydroxytamoxifen (4OH-TAM) [2,31]. As NDM-TAM is the primary metabolite regarding plasma concentrations, the demethylation followed by hydroxylation is supposed to be the main route [32].

## 3. Cytochrome P450 2D6

CYP2D6 is involved in the metabolism of ≈25% of the most commonly used drugs, whereas it only accounts for approximately 2% of total liver CYP protein capacity [2,33]. As shown in Figure 1, CYP2D6 is responsible for the specific conversion of NDM-TAM to endoxifen [31]. Therefore, CYP2D6 is considered the most important drug-metabolizing enzyme in tamoxifen metabolism. The *CYP2D6* gene is located on chromosome 22q13.2 and is highly polymorphic [34]. Currently, approximately 150 single nucleotide polymorphisms (SNPs) and 100 allelic variants are described [35]. Genetic polymorphisms may result in non-functional or reduced function alleles. Copy number variations, such as *CYP2D6* gene deletions and *CYP2D6* gene duplications, also occur [31,36]. This genetic variability results in individuals showing a broad spectrum of enzyme activities, indicated as poor (PM), intermediate (IM), normal (NM) or ultra-rapid metabolizers (UM) based on genetic composition [17,36]. Another approach is to use Activity Scores (AS), in which normal alleles are assigned a value of 1.0, decreased activity alleles are assigned a score of 0.5, and null alleles are assigned a score of 0.0 [17]. Genetic variants can be specific for certain populations and rarely found in other populations [33,37]. The variation in allele frequency results in differences in metabolic CYP2D6 activity amongst ethnic groups [33].

A translation of *CYP2D6* genotype into predicted CYP2D6 phenotype is shown in Table 1. Important changes were published in 2019, where it was internationally agreed to harmonize the CYP2D6*1/*4 interpretation from an Extensive/Normal metabolizer phenotype (CPIC definition until 2017, mostly used in US) into an Intermediate Metabolizer phenotype (definition used by the Dutch Pharmacogenetics Working Group (DPWG), which is mostly used in Europe). The second important change concerns the CYP2D6*10 allele, which was downgraded from AS = 0.5, comparable to other decreased activity alleles such as *9 and *41, to AS = 0.25 [38,39].

Most recently, Lee et al. suggested a dichotomization into normal and slow metabolizer CYP2D6 groups in an effort to improve and simplify the current system [40]. Nonetheless, these authors also recommend considering the direct measurement of endoxifen plasma concentrations, as *CYP2D6* genotype is not solely responsible for systemic endoxifen levels. In line with this recommendation, several authors [41,42,43,44] as well as the CPIC tamoxifen guideline [17] suggest that individualized dosing (e.g., therapeutic drug monitoring, TDM) might be a better option, instead of using a standard dosage of 20 mg tamoxifen per day. Another option is direct phenotyping, using dextromethorphan as a CYP2D6 phenotyping probe [45,46,47]. The advantage of this approach is that no genotype to phenotype translation is required, and that dose adjustment can be determined before the start of therapy [47]. Nevertheless, there are many more metabolites with unknown or estrogen-like properties [48]. Therefore, tamoxifen dosing solely based on (predicted) endoxifen blood concentrations might not be the best approach.

## 4. Influence of Other Drug Metabolizing Enzymes

In addition to CYP3A4/5 and CYP2D6, other CYP enzymes such as CYP2C9, CYP2C19, and CYP3A4 are involved in the tamoxifen metabolism. It is shown that genetic variation in these genes affects tamoxifen and metabolite plasma concentrations [49,50,51,52,53,54,55,56,57,58], although not all studies support these associations [59,60,61,62]. Several groups [59,61,62] examined the effect of variation in multiple CYP genes, but all concluded that only genetic variation in *CYP2D6* was associated with plasma concentrations of endoxifen.

The most important phase II enzymes in tamoxifen metabolism are uridine 5′-diphospho-glucuronosyltransferases (UGTs) and sulfotransferases (SULTs), primarily SULT1A1 [31,63]. Associations between genetic variation in UGTs or SULT1A1 and lower enzymatic activity [64], higher plasma levels of tamoxifen metabolites [65,66], or worse clinical outcome [66] were published. Therefore, these findings highlight the potential influence of UGTs and SULTs on tamoxifen disposition in breast cancer patients. In this light, the influence of the UGT inhibitor probenecid on tamoxifen metabolism is currently under investigation, and the results of this study are expected in 2021 (see Dutch Trial Registry: https://www.trialregister.nl/trial/8444, accessed on 28 November 2020). It is important to keep in mind that many enzymes may simultaneously influence the plasma levels of tamoxifen and its metabolites and therefore may also influence tamoxifen treatment efficacy.

## 5. Factors Affecting Endoxifen Levels

Madlensky et al. [67] reported in 2011 that patients with endoxifen levels >5.97 ng/mL (≈16 nM) had a 26% lower relative risk of breast cancer recurrence (hazard ratio (HR): 0.74; 95% confidence interval (CI) 0.55–1.00). In 2015, a comparable threshold for tamoxifen efficacy of plasma endoxifen at 5.2 ng/mL was described [50]. However, this threshold was questioned, because the study was not designed to determine this endoxifen threshold [68,69]. In 2020, in all homozygous carriers of *CYP2D6* non-functional alleles, but also in carriers of two reduced function alleles, significantly reduced endoxifen levels were observed [70]. A total of 118 Swedish premenopausal breast cancer patients genotyped for nine different allelic variants using blood as a source of genomic DNA showed that the endoxifen concentration in 32% of patients did not reach this putative threshold. Both NMs as well as UMs had endoxifen levels higher than the threshold of 5.9 ng/mL. Median endoxifen levels were 9.60 (interquartile range (IQR): 7.5–12.2) for normal or extensive metabolizers (NMs) and 12.8 (IQR: 9.7–16.0) ng/mL for ultra-rapid metabolizers (UMs). Poor metabolizers (PMs) and intermediate metabolizers (IMs) had endoxifen concentrations below this level (1.6–5.2 ng/mL). Interestingly, there was no statistical difference in endoxifen levels between PMs carrying two non-functional alleles and patients with the two decreased activity alleles *41/*41 or the combination *41/non-functional (defined as IM).

Nardin et al. [71] also confirmed in 2020 that tamoxifen metabolite levels were affected by the *CYP2D6* genotype, with the strongest effect for endoxifen concentrations. PMs carrying two non-functional alleles had 4.5 to 5.5 times lower endoxifen concentrations than NMs carrying two functional alleles. This study was conducted in 192 Brazilian breast cancer patients with endoxifen concentrations measured at 3, 6, and 12 months after the start tamoxifen treatment. Additionally, CYP2D6 phenotypes significantly predicted tamoxifen metabolite levels across all time points in multivariate analyses [71].

Taken together, these studies [70,71] confirmed that CYP2D6 metabolizer status is a strong determinant of endoxifen plasma concentration and that increasing CYP2D6 allele activity correlates with increasing plasma levels.

### 5.1. Tamoxifen Dose Escalation and Endoxifen Levels

Breast cancer patients with lower endoxifen levels than the published efficacy threshold of 5.9 ng/mL might benefit from a higher dose tamoxifen than the standard 20 mg/day. Khalaj et al. [72] performed a prospective clinical trial with a total of 134 Iranian ER-positive breast cancer patients. Patients with AS = 1 (*n* = 15) and AS = 0–0.5 (*n* = 2) received dose-adjusted therapy of 30 and 40 mg/day, respectively. Before dose escalation, the mean endoxifen concentration differed significantly between genetic phenotype groups, and a correlation between plasma endoxifen concentrations and CYP2D6 activity was observed, although considerable inter-individual variability within the metabolizer groups was present [72]. Dose adjustment in patients with AS = 0–1 also resulted in a significant increase in median endoxifen plasma concentrations, from 11.9 nM at baseline to 23.5 nM after 8 months [72]. This resulted in comparable endoxifen plasma concentrations as compared to patients with AS > 1 receiving the standard dose of 20 mg/day.

Tamura et al. [73] investigated the effect of *CYP2D6* genotype-guided tamoxifen dosing in a randomized, open-label, phase II study, in which 186 Japanese breast cancer patients were genotyped. Patients carrying at least one *CYP2D6* variant allele were randomly assigned to two groups: regular tamoxifen dosage (RD, *n* = 66, 20 mg/day) and increased tamoxifen dosage (ID, *n* = 70, 40 mg/day). This resulted in a significantly higher median serum through endoxifen concentration in the ID group as compared to the regular dose (RD) group [73]. In 2011, Irvin et al. [74] also demonstrated that dose escalation to 40 mg tamoxifen/day in American PM and IM breast cancer patients resulted in higher endoxifen plasma levels, as compared to PM and IM patients receiving the standard dose. The median plasma concentration increased by a factor of 1.2 in IMs and 3.1 in PMs. In 2015, Welzen et al. [75] performed a similar study in four PM and 12 IM breast cancer patients after tamoxifen dose adjustment from 20 to 40 mg/day. This showed an increment in median plasma concentration of endoxifen by a factor of 2.05 in PMs and by a factor of 1.83 in IMs. Nevertheless, after dose adjustment, there was still a significant difference remaining in endoxifen concentrations between NM and PM patients in both studies [74,75]. In 2015, Martinez de Dueñas et al. [76] showed similar levels of endoxifen concentrations in PM patients after dose escalation to 40 mg/day (*n* = 11) and subsequently to 60 mg/day (*n* = 8), and NM patients were treated with the standard tamoxifen dose. After increase of the dosage, no significant increase in the occurrence of the most common side effect (hot flashes) or severe side effects was found [72,73]. Meanwhile, the frequency of vomiting and agitation did increase, although only slightly [72]. This finding was in contrast with the previous studies [74,75,76], which reported no difference in the occurrence of side effects.

Currently, in the Netherlands, a large prospective clinical trial is ongoing in early stage ER-positive breast cancer patients, in order to assess the feasibility of TDM-guided dosing of tamoxifen in clinical practice (the TOTAM study). In this study, patients are genotyped for *CYP2D6* polymorphisms, and endoxifen concentrations are determined 3 months after the start of tamoxifen treatment (https://www.trialregister.nl/trial/6918, accessed on 28 November 2020). Patients with endoxifen levels below 16 nM (≈5.97 ng/mL) receive a dose adjustment of tamoxifen. Subsequently, endoxifen concentrations were monitored during a period of 2 years. The first positive results have recently been presented at the ESMO virtual congress 2020 and confirm that TDM-guided dosing is feasible in order to optimize endoxifen levels [77].

### 5.2. Patient Therapy Adherence and Endoxifen Levels

**Figure 2:**
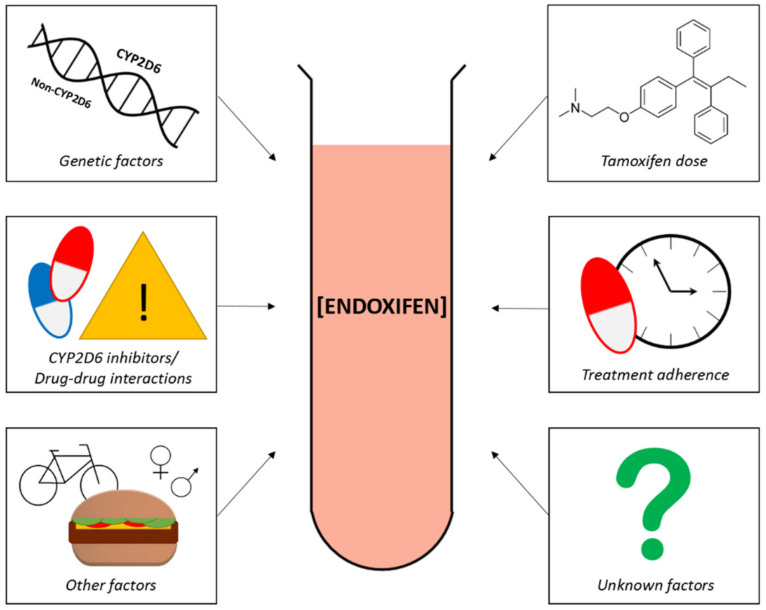
**Schematic overview of factors influencing plasma concentrations of endoxifen.** Known factors are genetic influences (e.g., *CYP2D6* polymorphisms but also non-*CYP2D6* polymorphisms), drug–drug interactions (e.g., concomitant use of CYP2D6 inhibitors), other factors (e.g., food, lifestyle, gender, age), tamoxifen dose, treatment adherence, and additional unknown factors.

The plasma concentration of endoxifen is affected by many factors (Figure 2). An important factor is treatment adherence. It is seen that breast cancer patients stop tamoxifen treatment before completing the standard treatment period of 5 years. Reasons for quitting early are the development of side effects, suboptimal patient physician communication, or low perception of the tumor recurrence risk [71]. Multivariate analyses showed that about 47% of tamoxifen plasma concentration variability is explained by patient adherence behavior; in addition, the combination of *CYP2D6* genotype and adherence behavior explained 40% of endoxifen plasma concentration variability at 12 months [71].

He et al. [78] investigated the relationship between CYP2D6 metabolizer status and tamoxifen discontinuation in a prospective–retrospective study, showing that UMs had a discontinuation rate of 18.8% at 6 months after the start of tamoxifen treatment. This was significantly higher than the discontinuation rate of NMs (6.7%) [78]. At the same time point, no significant difference in tamoxifen discontinuation was found for PMs (7.1%) or IMs (7.6%), as compared to NMs (6.7%) [78].

### 5.3. Drug–Drug Interactions

Drug–drug interactions (DDIs) represent one of the major confounding factors for the evaluation of drug efficacy, and their clinical relevance is not always thoroughly investigated [79,80]. As tamoxifen is co-prescribed with CYP2D6 inhibitors, as shown by Arafah et al. in 499 Saudi patients [81], it is of critical importance to understand if and how concomitant treatment with CYP2D6 inhibitors can influence tamoxifen and metabolite levels.

Borges et al. [82] prospectively enrolled 158 tamoxifen-treated patients, analyzed the *CYP2D6* genotype, and measured plasma concentrations of tamoxifen and its metabolites. It seems endoxifen plasma concentrations were significantly lower in CYP2D6 NMs with the concomitant use of (strong) CYP2D6 inhibitors as compared to those without the concomitant use of CYP2D6 inhibitors (23.5 ± 9.5 nM vs. 84.1 ± 39.4 nM, respectively) [82]. Similar results are shown if tamoxifen is concomitant used with selective serotonin reuptake inhibitors (SSRIs) [14]. In this study, endoxifen levels in NMs taking CYP2D6 inhibitors were 58% lower than in patients who did not use CYP2D6 inhibitors [14]. In a prospective trial, including patients treated with adjuvant tamoxifen, the influence of concomitant paroxetine use was studied [83]. Measuring plasma levels of tamoxifen and its metabolites in 12 women, before and after 4 weeks of paroxetine co-administration, showed significant lower endoxifen concentrations if tamoxifen is concomitantly used with paroxetine (from a mean of 12.4 ng/mL to 5.5 ng/mL) [83]. In addition, Binkhorst et al. [6] showed that switching patients using tamoxifen from a strong SSRI to a minor SSRI resulted in dramatically increased endoxifen concentrations. Monte et al. [84] showed in a prospective study that the co-administration of a CYP2D6-dependent probe drug (dextromethorphan) resulted in a 9.49 times higher chance of genotype–phenotype discordance based upon 3 h DX/DM ratio [84].

These studies indicate an important role of DDIs on tamoxifen activation. Treating physicians should take this into account in order to minimize deleterious interactions that may reduce drug efficacy [85].

Studies regarding the *CYP2D6* genotype and additional factors on plasma concentration endoxifen described in this section are summarized in Table 2.

## 6. *CYP2D6* Genotype and Outcome

The main goal of preemptive *CYP2D6* genotyping is improving tamoxifen treatment outcome. As summarized in 2009 by Dezentje et al. [86], studies published up to 2009 showed contradictory results. In 2018, a CPIC guideline on *CYP2D6* and tamoxifen was published [17], indicating an added value of *CYP2D6* genotyping prior to tamoxifen therapy as assessed by this international group of experts. However, in 2019, the European Society for Medical Oncology (ESMO) strongly argues against the use of the *CYP2D6* genotype for determining tamoxifen dosage in a clinical setting [18]. We reviewed the literature for publications from 2018 to 2021 on *CYP2D6* and clinical outcome to see if new information has arisen.

### 6.1. Positive Association CYP2D6 Genotype and Outcome

In 2018, Brooks et al. [87] showed that *CYP2D6* genotype was associated with breast cancer recurrence in patients treated with tamoxifen in an adjuvant setting. The *CYP2D6* genotype was determined in two groups of predominantly non-Hispanic white patients; 1514 contralateral breast cancer (CBC) cases and 2203 unilateral breast cancer controls. CYP2D6 NM patients with a first breast cancer and treated with tamoxifen had nearly a 40% lower risk of CBC compared to patients without tamoxifen treatment (AS ≥ 1, RR = 0.63; 95% CI 0.51–0.78). The risk of CBC in IMs and PMs (AS < 1) was not reduced by tamoxifen treatment (RR = 0.95 and RR = 1.18, respectively) as compared to IMs and PMs without tamoxifen treatment [87]. Despite a higher AS seemingly associated with a decreased recurrence risk of CBC, there was no significant difference in RR found between the metabolizer groups. The lack of information on *CYP2D6* copy number variations is an important limitation of this study; thereby, no identification of UMs was possible within the study population.

A prospective cohort study in 157 Egyptian metastatic breast cancer patients investigated the influence of *CYP2D6* polymorphisms on tamoxifen response after 6 months of tamoxifen treatment. Thirty carriers of *CYP2D6* variant alleles had progression within 6 months after the start of tamoxifen treatment, whereas fourteen patients achieved good clinical tamoxifen response after 6 months [88]. This suggests that patients with metastatic breast cancer carrying certain *CYP2D6* variant alleles have worse 6-month prognosis after tamoxifen treatment. A limitation of this study is the limited amount of allele variants (*n* = 4) genotyped, thereby increasing the chances of missing patients carrying non wild-type alleles. Tamoxifen is used as a treatment option for metastatic breast cancer instead of adjuvant therapy.

He et al. [78] examined the association between *CYP2D6* genotype and breast cancer prognosis in 1309 Swedish breast cancer patients treated with tamoxifen. HRs were calculated in order to determine the association between CYP2D6 metabolizer status and breast cancer-specific mortality. This resulted in an HR of 2.59 for PMs (95% CI 1.01–6.67), 1.48 for IMs (95% CI 0.72–3.05), 1 for NMs (reference), and 4.52 for UMs (90% CI 1.42–14.37) [78]. Thus, both PMs and UMs have a worse prognosis for breast cancer compared to NMs after receiving a standard dose of tamoxifen. Other studies already provided evidence that PMs have a lower endoxifen level than NMs; therefore, worse prognosis is expected [70,71]. Unlike PMs, UMs are expected to have a higher endoxifen level as compared to NMs and therefore should have a tamoxifen response after standard tamoxifen dosage. An explanation for the high HR for UMs might be higher endoxifen plasma concentrations, resulting in a higher frequency of adverse drug reactions (ADRs) and thereby higher discontinuation rates. It was shown that symptom-relieving drugs such as antinauseants, anxiolytics, and medications against hot flashes were more often used by UMs than by NMs [78]. Of note, no significant difference was found for PM and IMs compared to NMs for the use of symptom-relieving drugs. Additional analyses revealed that users of symptom-relieving drugs showed higher tamoxifen discontinuation rates [78]. There was a higher number of breast cancer mortality among patients who discontinued tamoxifen treatment [78]. Due to the low proportion of UMs in the population, the study was underpowered to determine underlying causes of higher breast-cancer specific mortality rates in this specific group.

### 6.2. No Association CYP2D6 Genotype and Outcome

In 2019, Sanchez-Spitman et al. [89] conducted a prospective clinical study in which 667 Dutch or Belgian breast cancer patients treated with adjuvant tamoxifen were enrolled. After *CYP2D6* genotyping, patients were classified into five different groups; UMs, NMs, hetEM (heterozygous EMs), IMs, and PMs. Patients with two fully active alleles were classified as NM, whereas patients with one fully active and one non-functional allele were classified as hetEM. The 3-year relapse-free survival (RFS) was compared between the combined group of PMs, IMs, and hetEMs (*n* = 316), and the combined group of NMs and UMs (*n* = 322). Univariable analysis showed no difference in RFS between the two combined groups [89]. Classification, either based on the suggested threshold of 5.9 ng/mL or by quartile, did not result in any significant association between endoxifen levels and RFS [89]. This study received criticism by various researchers. Goetz et al. [90], Brauch et al. [91], and Braal et al. [92] stated that there were major issues regarding study design and statistical power. Firstly, patients included in this study received additional systemic therapy or switched to aromatase inhibitors instead of tamoxifen monotherapy in adjuvant setting. This may alter hazard for both early and late breast cancer events [90]. In addition, the anticipated effect size of HR = 2.0 at 3 years was said to be overestimated, since studies with a longer clinical follow-up of 5 and 10 years were at the basis for this HR [91]. Moreover, the study was not powered to investigate the relationship between endoxifen concentrations and clinical outcome [92]. Gusella et al. [47] stated that given the long time to recurrence of breast cancer, the short therapy duration and short follow-up might explain the negative results of this study. Moreover, the differences between the individual phenotype groups were not investigated. Of note, according the CPIC guidelines, hetEMs should have been classified as IMs; however, since hetEMs and IMs were combined in the same subgroup, this misclassification had a limited impact. A lack of impact of *CYP2D6* genotype on breast cancer-free survival (BCFS) was also reported by Rangel-Méndez et al. [93]. In this retrospective study, in 71 Mexican Mestizo patients receiving adjuvant tamoxifen treatment (20 mg/day), there was no statistical difference found in BCFS and recurrence risk between the NM/UM group (*n* = 56) and PM/IM group (*n* = 15) [93]. The limited sample size may have hindered the ability to find a significant association in this study [93].

Hertz et al. [94] conducted a retrospective PGx analysis based on 469 ER-positive Caucasian breast cancer patients treated with adjuvant tamoxifen monotherapy to investigate the association between low-activity *CYP2D6* genotype and recurrence-free survival (RFS). Patients with AS = 0 (PMs) and patients with AS > 0 (IM, NM, and UM) were compared; however, no association of *CYP2D6* status with RFS was found. After adjustment for relevant clinical covariates, analyses showed that a higher AS was associated with inferior RFS (HR: 1.43; *p* = 0.05). In 476 breast cancer patients who did not receive adjuvant tamoxifen therapy, an AS > 0, as well as an increasing AS, were associated with superior RFS (HR = 0.41 and HR = 0.66, respectively; *p* = 0.0015).

Tamura et al. [73] examined the effect of *CYP2D6* genotype-guided tamoxifen dosing on progression-free survival (PFS) in 186 Japanese breast cancer patients. The PFS rates at 6 months did not differ between the increased dosage (ID) group (67.6%) and the regular dose (RD) group (66.7%). PFS curves of both arms did not significantly differ [73]. The 6 months PFS rate might not be adequate to assess the response by endocrine treatment of ER-positive metastatic breast cancer. Additionally, in this study, but also in the study of Sanchez-Spitman et al. [89] and Rangel-Méndez et al. [93], they did not make a distinction between non-functional and reduced function *CYP2D6* variant alleles. This resulted in combining PMs and IMs in the intervention arm.

Publications from 2018 to 2021 on *CYP2D6* and clinical outcome described in this section are summarized in Table 3.

## 7. Conclusions

CYP2D6 is the main enzyme in the conversion of the prodrug tamoxifen into its most important metabolite endoxifen. Currently, a plasma concentration endoxifen of 5.97 ng/mL is maintained to indicate the sufficient efficacy of tamoxifen treatment. Patients with reduced CYP2D6 activity due to genetic variations often fail to reach this limit. Tamoxifen dose increments in these CYP2D6 compromised patients result in higher plasma concentrations of endoxifen without a higher frequency of ADRs. In addition to the *CYP2D6* genotype, additional factors also seem to influence endoxifen levels, such as treatment adherence and drug–drug interactions, for example caused by the concomitant use of (strong) CYP2D6 inhibitors. In the last years, various prospective and retrospective studies were performed to examine the association between *CYP2D6* genotype and prognosis in (ER-positive) breast cancer patients, which led to, again, contradictory results. Some of these studies have been criticized, mainly because they were neither designed nor statistically powered to answer specific pharmacogenetic questions. Between 2018 and 2021, two studies provided evidence for a relation between *CYP2D6* genotype and breast cancer recurrence as well as breast cancer-specific mortality after tamoxifen treatment. In that same period, four studies reported no association with clinical outcome in tamoxifen treatment. Therefore, it seems that the controversy regarding the association of *CYP2D6* genotype and tamoxifen-related clinical outcome continues. In our opinion, this controversy will exist up until large randomized control trials will be performed.

## Figures and Tables

**Table 1:** Adapted final consensus *CYP2D6* genotype to phenotype table. Combining the previous CPIC and DPWG guidelines and adding new pharmacogenetic insights [38]. Abbreviations: CYP2D6: Cytochrome P450 2D6, UM: ultra-rapid metabolizer, NM: normal or extensive metabolizer, IM: intermediate metabolizer, PM: poor metabolizer, CPIC: Clinical Pharmacogenetic Implementation Consortium, DPWG: Dutch Pharmacogenetics Working Group.

Likely Phenotype	CURRENT CPIC Activity Score Definition	CURRENT DPWG Activity Score Definition	NEW Standardized Activity Score Definition
*CYP2D6 UM*	>2	>2.5	>2.25
*CYP2D6 NM*	1–2	1.5–2.5	1.25–2.25
*CYP2D6 IM*	0.5	0.5–1.0	0.25–1.0
*CYP2D6 PM*	0	0	0

**Table 2:** Summary of described recent studies on *CYP2D6* genotyping, and additional factors on plasma concentrations endoxifen.

Reference	End-Point	N. PTS.	Material and Methods	Results
Thorén et al., 2020 [70] Nardin et al., 2020 [71]	Endoxifen plasma concentrations in PM, IM, NM, and UM patients	118, 192	*CYP2D6* genotyping; LC-MS/MS	CYP2D6 metabolizer status is a strong determinant of plasma endoxifen concentrations. Increasing *CYP2D6* allele activity correlates with increasing endoxifen levels.
Khalaj et al., 2019 [72] Tamura et al., [73]	Endoxifen plasma concentrations in PM, IM, NM, and UM patients	134, 186	*CYP2D6* genotyping; LC-MS/MS	Dose escalation in CYP2D6-compromised patients (PMs and IMs combined) resulted in an increase in endoxifen levels, similar as NMs (using standard dosage of 20 mg/day). No difference in the occurrence of the most common side effect (hot flushes) or severe side effects was found.
Nardin et al., 2020 [71]	Endoxifen plasma concentrations in PM, IM, NM and UM patients; Patient adherence behavior	192	*CYP2D6* genotyping; LC-MS/MS; Morisky, Green, and Levine medication adherence scale	Adherence explained 47% of tamoxifen variability (*p* < 0.001). Combination of patients adherence and *CYP2D6* genotype explained 40% of endoxifen variability at 12 months (*p* < 0.001). So, endoxifen levels are influenced both by patients’ tamoxifen treatment adherence and *CYP2D6* genotype.
He et al., 2020 [78]	Tamoxifen discontinuation	1309	*CYP2D6* genotyping; Self-reported questionnaires	UMs show a significantly higher discontinuation rate at 6 months after start of tamoxifen treatment (18.8%) compared to NMs (6.7). No significant difference in tamoxifen discontinuation was found for PMs (7.1%) or IMs (7.6%). After 6 months, no significant difference in discontinuation rates was found.
Monte et al., 2018 [84]	Dextro-methorphan (DM)/dextror-phan (DX) ratio in PM, IM, NM, and UM patients	39	*CYP2D6* genotyping; Plasma DM and DX assay using LC-MS	Patients with co-ingestion of dextromethorphan (as CYP2D6 enzyme probe drug) and another CYP2D6-dependent drug were 9.5 times more likely to have genotype-phenotype discordance based upon the 3 h DX/DM ratio.

**Table 3:** Summary of described recent studies on *CYP2D6* genotyping in relation to tamoxifen-related clinical outcome.

Reference	End-Point	N. PTS.	Material and Methods	Results
Brooks et al., 2018 [87]	Recurrence risk of contralateral breast cancer	1514 cases, 2203 controls	*CYP2D6* genotyping; population based case-control study.	No significant difference in recurrence risk between metabolizer groups (*p* = 0.09). However, a trend of decreasing CBC RR associated with tamoxifen treatment, with increasing AS was shown.
Malash et al., 2020 [88]	Frequency of *CYP2D6* variant alleles in responder vs. refractory group	157	*CYP2D6* genotyping; Patient assessment for adverse events and tumor response after 4, 8, 16 and 24 weeks	Wild-type *CYP2D6* was present in 113 of the patients; 62.0% in the refractory group and 82.1% in responders. In 44 patients, *CYP2D6* polymorphisms were detected: 30 of them were refractory (68.2%) and 14 were responders (31.8%). CYP2D6*3 and *4 were the most common *CYP2D6* variants detected in the refractory group (86.7%). CYP2D6*10/*10 and *10/*3 were the most common variant diplotypes (85.7%) in the responders group.
He et al., 2020 [78]	Breast cancer-specific mortality	1309	*CYP2D6* genotyping;	Patients genotyped as PM and UM show worse prognosis compared to NMs under standard tamoxifen dosage (20 mg/day) (HR: 2.59, 95% CI 1.01–6.67 and HR: 4.52, 95% CI 1.42–14.37 respectively).
Sanchez-Spitman et al., 2019 [89]	Relapse-free survival	667	*CYP2D6* genotyping; prospective CYPTAM study using Cox regression analysis.	No significant difference in RFS between combined groups of PMs + IMs + hetEMS and NMs + UMs (*p* = 0.944). *Of note: This study was seriously criticized for several reasons.*
Rangel-Méndez et al., 2020 [93]	Breast cancer-free survival	71	*CYP2D6* genotyping; retrospective study, using Kaplan–Meier method and log-rank test to estimate BCFS	No difference in BCFS and recurrence risk between combined group of NMs + UMs and PMs (*p* = 0.45) and IMs (*p* = 0.55).
Tamura et al., 2019 [73]	Progression-free survival rate at 6 months	186	*CYP2D6* genotyping; A randomized, open-label, multicenter, phase II study.	No significant difference in PFS rate at 6 months between increased dosage arm and regular dosage arm (67,6% vs. 66,7%). Survival curves did not significantly differ (*p* = 0.15).

## Data Availability

Not applicable.
